# Exonuclease activity and P nucleotide addition in the generation of the expressed immunoglobulin repertoire

**DOI:** 10.1186/1471-2172-5-19

**Published:** 2004-09-02

**Authors:** Katherine JL Jackson, Bruno Gaeta, William Sewell, Andrew M Collins

**Affiliations:** 1 School of Biotechnology and Biomolecular Sciences, University of New South Wales, Sydney, Australia; 2 Garvan Institute of Medical Research, Sydney, Australia; 3 St Vincent's Clinical School, University of New South Wales, Sydney, Australia

## Abstract

**Background:**

Immunoglobulin rearrangement involves random and imprecise processes that act to both create and constrain diversity. Two such processes are the loss of nucleotides through the action of unknown exonuclease(s) and the addition of P nucleotides. The study of such processes has been compromised by difficulties in reliably aligning immunoglobulin genes and in the partitioning of nucleotides between segment ends, and between N and P nucleotides.

**Results:**

A dataset of 294 human IgM sequences was created and partitioned with the aid of a probabilistic model. Non-random removal of nucleotides is seen between the three IGH gene types with the IGHV gene averaging removals of 1.2 nucleotides compared to 4.7 for the other gene ends (p < 0.001). Individual IGHV, IGHD and IGHJ gene subgroups also display statistical differences in the level of nucleotide loss. For example, within the IGHJ group, IGHJ3 has average removals of 1.3 nucleotides compared to 6.4 nucleotides for IGHJ6 genes (p < 0.002). Analysis of putative P nucleotides within the IgM and pooled datasets revealed only a single putative P nucleotide motif (GTT at the 3' D-REGION end) to occur at a frequency significantly higher then would be expected from random N nucleotide addition.

**Conclusions:**

The loss of nucleotides due to the action of exonucleases is not random, but is influenced by the nucleotide composition of the genes. P nucleotides do not make a significant contribution to diversity of immunoglobulin sequences. Although palindromic sequences are present in 10% of immunologlobulin rearrangements, most of the 'palindromic' nucleotides are likely to have been inserted into the junction during the process of N nucleotide addition. P nucleotides can only be stated with confidence to contribute to diversity of less than 1% of sequences. Any attempt to identify P nucleotides in immunoglobulins is therefore likely to introduce errors into the partitioning of such sequences.

## Background

The variable domain of the immunoglobulin heavy chain (IGH) is encoded by the IGHV (variable), the IGHD (diversity) and the IGHJ (joining) genes. In developing B cells these genes are brought together via a process of recombination involving the selection of one of each gene type from sets of genes present within the genome [1]. The bringing together of the selected IGHV, IGHD and IGHJ genes generates combinatorial diversity [1]. The first genes to join are the IGHD and IGHJ genes, followed by the bringing together of the IGHV gene with D-J. Further junctional diversity is generated at the points between the joining genes [2,3]. Junctional diversity results from the loss of nucleotides through the action of unknown exonuclease(s) and from the addition of N [3] and P nucleotides [2]. The final IGH V-D-J rearrangement in mature B cells is finally subject to the process of somatic hypermutation in secondary lymphoid organs which involves the targeted introduction and accumulation of point mutations [4].

The addition of N nucleotides is performed by the enzyme terminal dideoxynucleotidyl transferase (TdT), and in the IGH locus this addition can occur at both the D to J and the V to D-J joins [5]. The regions of N addition are denoted as N regions, and nucleotides that fall between the V and D genes are denoted as N1 regions, while those that lie between the D and J genes are denoted as N2 regions.

P nucleotides are derived from the asymmetric opening of hairpin loops that form at gene ends as part of the rearrangement process [6]. The opening of the hairpin loops produces short, self-complementary single stranded extensions that can be incorporated into junctions, or may alternatively be removed via exonuclease activity [6]. It is the self-complementarity of P nucleotides that leads to their palindromic appearance and thus to their name. Hairpin opening is said to produce inserts of 0–4 nucleotides [2]. P nucleotides have been associated with the IGHV and IGHJ genes, as well as with each end of the IGHD gene [7] and estimates of the frequency of P nucleotide addition suggest a presence in about ten percent of sequences [7-10].

The mechanism of immunoglobulin gene rearrangement was first proposed by Tonegawa in the late 1970's [1]. Since that time, much has been learnt about the processes involved. Some areas, however, remain relatively uninvestigated, including the nature of exonuclease removal and the contribution of P nucleotide addition to junctional diversity. The lack of research in these fields may reflect the inherent difficulties in studying the relevant gene sequences, because IGH V-D-J junctions are the result of random and imprecise processes. It can therefore be difficult to distinguish between gene ends and N or P additions.

The very few reports of exonuclease removal in the literature mainly describe analysis of murine sequences [11-14]. These investigations revealed nucleotide loss to be significantly different for murine IGHJ and IGHD genes. Differences were seen in the average exonuclease removal from IGHJ and IGHD gene subgroups, with individual gene subgroups possessing significantly different average levels of nucleotide removal. Influences upon gene processing that have been proposed to explain these observations include the presence of TG motifs [15], the relative location of stretches of 3 or more W (A or T) nucleotides and their positional relationship with respect to 2 or more S (G or C) nucleotides [12], and the presence of TAT motifs [13].

Recent advances in data standardisation in immunogenetics has allowed for improved statistical analysis. The standardisation emanates from IMGT-ONTOLOGY [16,17] upon which one of the most widely used immunogenetics tools, IMGT/V-QUEST, is based [18]. IMGT, the IMmunoGeneTics Information System^*R*^, also offers standardised nomenclature [19] and standardised numbering of positions within immunoglobulin sequences [20]. Despite the importance of IMGT within the field, the tools offered still suffer from shortcomings especially in the analysis of IGH V-D-J junctions. Alternative means of analysing junctions are therefore still sought by researchers [21,22].

The development of a statistically based algorithm for the partitioning of immunoglobulin sequences [21] as an alternative to IMGT/Junction Analysis [18], combined with the large amount of sequence data available through public nucleotide databases, has allowed us to investigate the nature of nucleotide removal from human immunoglobulin heavy chain genes in the expressed repertoire. Improved means of identification of gene ends facilitates the development of datasets with more certain partitioning. This study reports the extent of P nucleotide addition and the nature of exonuclease removal in the expressed human repertoire. Analysis of nucleotide loss and addition within the dataset reveals that different gene subgroups undergo distinct processing by exonuclease(s) and shows that there is no significant contribution by P nucleotides to the diversity of the expressed repertoire.

## Results

### Dataset creation

The collection of human IgM sequences from public databases resulted in a dataset of approximately 1500 sequences. The exclusion of fetal, moderately and highly mutated (>5 mutations) and disease associated sequences reduced the dataset to 306 sequences. Further exclusions were made of those sequences that showed signs of IGHV gene replacement or the utilization of multiple D genes. Five sequences utilized two D gene segments, as identified using strict criteria as previously described [21] (EMBL:U97246, L12190, L29154, AJ519292, AJ245025). Evidence of IGHV gene replacement, in the form of V gene 'footprints', was seen in 7 sequences (EMBL:L29154, AJ245008, AJ245280, AJ519296, AY003831, X54445 Kabat:AL311). The footprints were unique sequences of 6 or more nucleotides derived from V gene ends containing a cryptic recombination signal sequence (cRSS) which is thought to be essential for replacement events [23-26]. The final dataset contained 294 sequences.

Within the final dataset of 294 sequences there were 245 sequences for which IGHV, IGHD, IGHJ, N1 and N2 regions could be defined. A further 49 sequences, lacking determinable D, N1 and N2 region but possessing identifiable IGHV and IGHJ genes were also included in the dataset. For these 49 sequences, it was not possible to confidently determine the utilized IGHD gene within the junctional nucleotides, however, the ends of the V and J regions could be determined accurately. All IGHV, IGHD and IGHJ gene subgroups were represented within the dataset. Details of the dataset can be seen in the Appendix [see Additional File 1].

### Exonuclease removals from genes and gene subgroups

**Figure 1:**
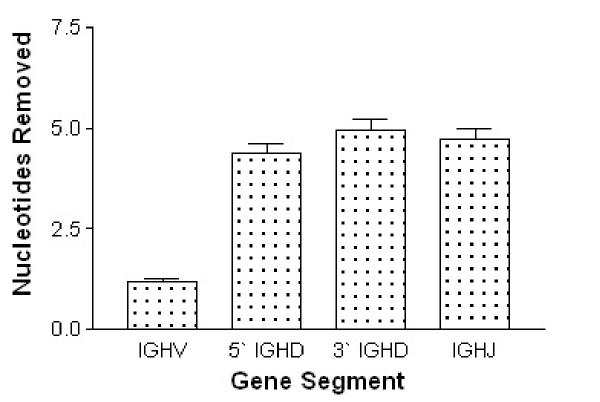
**Average exonuclease removal from IGH genes. **The average nucleotide removal from of the gene ends was examined for 294 IGHV and IGHJ genes and 245 IGHD genes. For the IGHD genes, removals were considered from each end of the gene; 5' (V-D side) and 3' (D-J side). Bars represent standard error.

Exonuclease removal was evident in each of the 245 IGH V-D-J rearrangements examined, with 25% of IGH V-D-J rearrangements displaying removal from all four gene ends. A further 48% of IGH V-D-J sequences had removals from three of the four gene ends. The average number of nucleotides lost from each of the gene ends is presented in Figure 1.

Examination of the 294 IGHV and IGHJ segments revealed 41% of IGHV ends lacked removals, compared to just 18% of IGHJ ends. Sixteen percent of the 245 IGHD genes lacked removals from the 5' end of the D gene, and 17% had no removals from the 3' end of the D region. Exonuclease removals ranged from 0 to 13 nucleotides at the 3' V-REGION and 0 to 14 nucleotides at the 5' D-REGION end. At the D-J junction, 3' D-REGION and 5' J-REGION removals both ranged from 0 to 20 nucleotides.

A significant difference in the extent of exonuclease removals was observed between the IGHV, IGHD and IGHJ gene ends (p < 0.0001, Kruskal-Wallis Test). Average removals from IGHV region ends were significantly lower than removals from IGHD and IGHJ region ends (p < 0.001, Dunn's Multiple Comparison Test). On average, only 1.2 nucleotides were lost from IGHV region ends while average removals of 4.7 nucleotides were evident from each of the IGHD region ends as well as from the IGHJ region ends.

**Figure 2:**
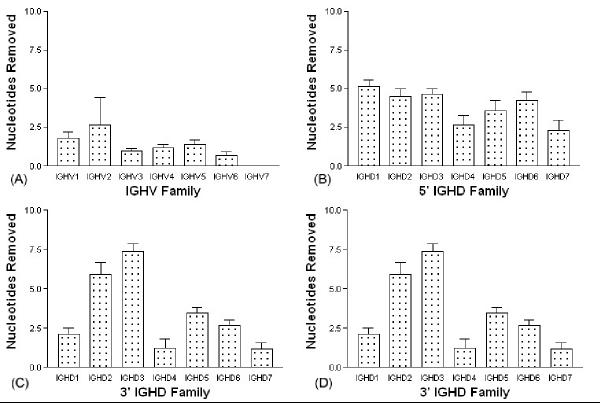
**Average exonuclease removal from gene subgroups. **The average exonuclease removals from gene ends was investigated for each IGHV, IGHD and IGHJ subgroup. Significant differences were seen among the 294 IGHV genes (A). No significant difference between the 245 IGHD genes were seen at the 5' end (B). The 3' IGHD end does show significant differences for the IGHD subgroups (C) as do the six IGHJ subgroups (D). Bars represent standard error.

The average number of nucleotides removed from each gene subgroup within the three genes was calculated to identify differences in processing of sequences at the gene subgroup level (Figure 2). It was necessary to exclude IGHV7 from the analysis as only a single sequence from this subgroup was in the dataset. Average removals differed significantly among the six IGHV subgroups analyzed (p = 0.03, Kruskal-Wallis Test) (Figure 2A). Comparison testing was, however, unable to identify the source of the difference.

Removals from D gene subgroups were examined at each of the region ends. The removals from the 5' D end did not reveal significant differences, but significant differences were seen between the subgroups at the 3' end (p < 0.0001, one-way ANOVA) (Figure 2B, Figure 2C). More extensive removals, of 6.0 and 7.5 nucleotides respectively, were observed from IGHD2 and IGHD3 subgroup members (p < 0.0001, Tukey's Multiple Comparison Test). The remaining 5 IGHD subgroups experienced average deletions of 2.1 nucleotides at their 3' ends.

Comparison of average nucleotide loss for each of the six IGHJ subgroups revealed significant differences between the average removals (p < 0.0001, one-way ANOVA). The low level of removals from IGHJ3 was notable. On average just 1.3 nucleotides were removed from IGHJ3 sequences, while an average of 6.4 nucleotides were removed from IGHJ6 sequences (p < 0.002, Tukey's Multiple Comparison Test) (Figure 2D).

### Influence of W and S motifs

**Figure 3:**
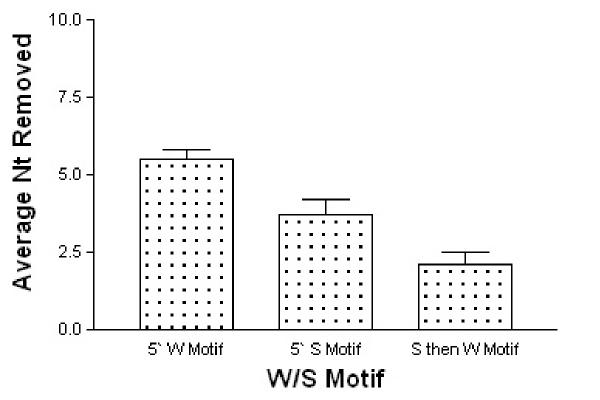
**Influence of W and S motifs on nucleotide loss. **IGHJ genes were grouped by the presence of W and S motifs within the first 15 nucleotides of the IGHJ subgroup sequence. Average exonuclease loss was examined for the three sets; 5' W only, 5' S only and S then W.

A more detailed examination of exonuclease removals from the IGHJ genes was undertaken, to investigate the influence of W and S motifs. The presence of these motifs in the first 15 5' nucleotides of the IGHJ ends was considered. IGHJ ends containing 5' S motifs showed significantly lower average removals than those lacking a 5' S motif (p < 0.0001, Kruskal-Wallis Test). The IGHJ genes whose sequences did not possess an S motif within the first 15 5' nucleotides had, on average, three more nucleotides removed (Figure 3).

### Contribution of P nucleotides to diversity

Putative P nucleotides were identified among those gene ends that remained untrimmed by exonuclease activity during the process of IGH V-D-J rearrangement. Examples of nucleotides that satisfied the P nucleotide criteria were observed at 3' V, 5' D, 3' D and 5' J gene ends. The identified nucleotides ranged in length from 1 to 4 nucleotides. The observed P nucleotides fell into twenty-three sets based upon unique sequences, and the gene end at which they were observed (Table 1). Each of the 23 sets was analyzed to determine the likelihood that apparent P nucleotides were actually the result of N additions. The p-values for each of the P nucleotide sequences are shown in Table 1. Correction of the significance level for the comparison of the 23 sets using the Bonferroni adjustment resulted in a required alpha value of 0.003.

**Table 1:** Putative P Nucleotides in a dataset of 294 human IgM sequences

Gene Segment	Putative P Sequence	Observed	Total Junctions	p-value^1^
IGHV	C	10	111	0.99
	T	25	111	0.023
	CC	2	105	0.99
	TC	10	105	0.049
	TG	2	105	0.98
	TCT	3	100	0.045
	TGT	1	100	0.55
	TCTC	1	86	0.21
IGHD 5'	A	1	37	0.99
	C	5	37	0.99
	AC	1	36	0.86
	CA	1	36	0.86
	CC	2	36	0.96
	CCC	1	33	0.77
IGHD 3'	G	11	40	0.88
	GT	2	36	0.57
	TC	1	36	0.86
	GTT	3	33	0.0022
IGHJ	T	11	42	0.042
	GT	3	34	0.26
	AGC	1	32	0.45
	ACT	2	32	0.026

^1 ^α equal to 0.003

A single case of significance was observed among the putative P nucleotide sequences. This was for a sequence of 3 nucleotides (GTT) which was associated with the 3' end of the IGHD region. The occurrence of 3 'GTT' sequences in the dataset remains the only significant putative P nucleotides even if the alpha value is increased to 0.01. Using a 0.05 significance level, 6 sets out of 23 appear significant, however this conclusion carries a 69% chance of being incorrect and that the results occurred by chance.

Although putative P nucleotide sequences are present in 10% of sequences most of these are likely to have arisen as the result of N nucleotide addition. P nucleotides can only be confidently attributed to less than 1% of sequences with three sequences from the IgM dataset contained statistically significant P nucleotides out of the 245 IGH V-D-J rearrangements examined. The overall contribution of P nucleotides to junctional nucleotides was 9 nucleotides out of 2899 junctional nucleotides, or 0.3% of junctional nucleotides, within the IgM dataset. The probability of 'GTT' occurring within an N region is 0.007875, therefore, the sequence could be expected to occur twice at the observed position in the 245 junctions examined, by chance alone. Of the three identified P nucleotides it is therefore possible that only one is a true P nucleotide. The contribution of P nucleotides to junctional nucleotides could therefore be as low as 0.1%, with P nucleotide inclusions occurring in less than 0.5% of sequences.

## Discussion

Investigation of the role played by nucleotide loss and addition in the generation of immunoglobulin diversity has been limited by the ability of researchers to accurately determine gene ends. The development of a statistically based partitioning method has allowed this study to gain insights into the nature of nucleotide loss and addition in the expressed human immunoglobulin repertoire. Analysis of 294 human IgM sequences revealed significant differences between average nucleotide losses from different heavy chain genes segments. IGHV genes suffer less removal in comparison to other genes, suggesting that a process or processes may act to prevent the removal of critical components or to select against sequences in which such removals have occurred. Critical components may include the conserved TGT that defines the start of the CDR3 [27] and the internal heptamer site utilized in V_*H *_gene replacement [23-25].

The extension of the analysis to the gene subgroup level showed significant differences among removals from the IGHV gene subgroups, among the IGHD subgroups at the 3' end of the D gene and between the six IGHJ gene subgroups. The most striking contrast was observed for the IGHJ gene subgroups, specifically for IGHJ3 and IGHJ6. Removals from IGHJ3 averaged only a single nucleotide, while IGHJ6 on average lost in excess of 6 nucleotides. The differences observed as part of this study suggest that the loss of nucleotides during the creation of human heavy chain immunoglobulin sequences is not random.

Unique 'patterns' of exonuclease removal between gene subgroups have previously been reported in murine immunoglobulins [12,28], however we are not aware of any such reports from studies of the human repertoire. Murine IGHJ4 genes have been reported to undergo an average removal of 2 nucleotides more than any other murine IGHJ subgroup [28]. Comparison of the murine IGHJ4 sequence to that of human IGHJ6 shows these two sequences to be identical for the first 7 nucleotides. The common sequence, ATTACTA, is unique to these IGHJ subgroups. This suggests that the common sequence may be linked to the high levels of nucleotide loss experienced by these gene subgroups, relative to the other IGHJ genes.

The nucleotide composition of gene has previously been stated to influence exonuclease processing in the murine system [12]. The presence of two 'motifs' was thought to be the determining factor in the outcome of exonuclease processing. One motif involves stretches of two or more G or C nucleotides and is referred to here as the S motif. The other motif is composed of stretches of three or more consecutive A or T nucleotides and is referred to here as the W motif. S motifs in murine sequences were associated with low average removals from gene region ends, while the presence of W motifs correlated with high average removals [12]. Similar results were seen for human immunoglobulins in this study, with average removals from IGHJ genes containing 5' S motifs being significantly lower than from those containing 5' W motifs. Interestingly, the average position of the first S motif within the IGHJ genes coincided with the average level of removal from IGHJ genes. The correlation between S motif position and average exonuclease removal suggests that the S motif may act to block continued exonuclease removal from the gene region end. This may explain the high removals from the human IGHJ6 and murine IGHJ4 gene segments, as these sequences lack S motifs which may prevent such extensive exonuclease processing.

Consideration of the W and S motif composition of the 3' D gene segments showed the IGHD2 and IGHD3 gene subgroups to be rich in W motifs and to lack S motifs (data not shown). These two subgroups showed higher average removals compared to other IGHD subgroups. The relationship between nucleotide composition and exonuclease activity could therefore explain the significant differences observed at the 3' end of the IGHD gene subgroups.

IGHD2 and IGHD3 are both long D genes. A relationship between D gene length and exonuclease activity may have therefore been acting to influence exonuclease processing. Examination of exonuclease activity of D genes grouped by length did reveal significant differences (data not shown), however, these differences were only evident at the 3' end of the D genes and as the analysis was confined to IGHD2 and IGHD3 sequences, it is difficult to conclude whether sequence length has a role.

The influence of nucleotide composition on exonuclease removals from heavy chain gene is easily examined in the IGHJ genes, due to the small number of alleles and the clear division of sequences based on the presence or absence of S and W motifs. Significant differences in the V genes were not further examined as the larger number of alleles made sample groups too small to allow for meaningful statistical analysis. The absence of significant differences at the 5' end of the IGHD genes may result from the lack of distinct differences in the nucleotide composition of these sequences. This would make any variations in exonuclease processing more subtle, and thus a larger sample size would be necessary to observe any differences.

P nucleotide addition has been reported to contribute to diversity in between 10% [7,8,29] and 41% [9] of immunoglobulin sequences. Initial analysis of putative P nucleotides in the dataset of 294 human IgM sequences in this study revealed the frequency the presence of putative P nucleotides to be around ten percent of sequences. This is consistent with previous reports [7,10,29]. Statistical analysis of the putative P nucleotides sequences, however, revealed that only one 'P nucleotide sequence' was observed at a frequency that was significantly above the frequency that would be expected from N nucleotide addition alone. This suggests that the true contribution of P nucleotides to diversity in the expressed human IgM repertoire is much lower than previously reported, with P nucleotides present in less than 1% of sequences and accounting for approximately 0.3% of junctional nucleotides in the IgM dataset.

It should be noted that even those P nucleotides accepted on the basis of statistical analysis carry a degree of uncertainty. Accounting for the possible misidentification of TdT additions among P nucleotides suggests that the contribution of P nucleotides to junctional nucleotides may be even lower than 0.3%. The identification of P nucleotides during partitioning of immunoglobulin sequences introduces a greater margin of error than would result from their exclusion from partitioning processes. For example, the rare nature of P nucleotide inclusion in rearranged immunoglobulins means that in a dataset of 1000 sequences less than 10 sequences may possess P nucleotides. Arbitrary identification of putative P nucleotides in the same dataset could however lead to 100 of the sequences being identified as having P addition. The error is therefore smaller if P nucleotides are not allocated as part of the partitioning process.

The statistical demonstration of the phenomenon of P nucleotides by Meier and Lewis utilized altered recombination substrates, where the IGHV, IGHD and IGHJ gene regions were replaced by restriction sites on a plasmid vector [8]. The recombinant substrates were then transfected into murine cell lines and the processing of the substrates was then examined. Meier and Lewis observed that putative P nucleotides occur at a significant frequency among the processed recombinant substrates and this has been used as the basis for the allocation of P nucleotides in subsequent immunoglobulin studies [8]. Examination of the frequency of P nucleotides among the recombination substrates, however, revealed a five fold greater frequency of P nucleotide inclusions than was seen among adult murine T cell receptors and immunoglobulin sequences [8] and adult human immunoglobulins [7,29]. The applicability of Meier and Lewis' statistical analysis of the altered recombination substrates to demonstrate the contribution of P nucleotides must therefore be questioned, as the reporting of statistical significance is likely to be a direct result of the elevated frequency of putative P nucleotides among the recombinant substrates. Examination of putative P nucleotides at five-fold lower frequencies eliminates the significance observed in the original studies and supports the figure reported here of a contribution to junctional diversity in less than 1% of sequences (data not shown).

## Conclusions

Substantial *in vitro *evidence in support of the formation of hairpin loops as part of the immunoglobulin rearrangement mechanism [6,30,31] and for the creation of P nucleotides as part of the process of hairpin loop opening exists [32,33]. The results reported here suggest that the P nucleotides generated by the loop opening do not, however, contribute significantly to the diversity of the final rearranged immunoglobulin. Exonuclease processing of IGH genes is not random and the nucleotide composition of the gene end appears to be influential. Further investigations into factor(s) influencing the exonuclease processing of gene ends will be required in order to elucidate the exact nature of the relationship between the gene end and exonuclease processing.

## Methods

### Dataset creation

Human IgM sequences were obtained from public nucleotide databases; IMGT/LIGM-DB available through the IMGT, The IMmunoGeneTics Information System^*R*^, [18], the Entrez nucleotide database from the National Center for Biotechnology Information (NCBI) [34] and the Kabat Database of Sequences of Immunological Interest [35]. The sequences obtained were screened to exclude those sequences of fetal origin, those associated with diseases and those of a non-productive nature. Screening was necessary to avoid the introduction of any biases that may be associated with particular disease states or stages of immunological development. Sequences that contained in excess of 5 mutations within the V gene were also excluded from the final dataset. Sequences displaying higher levels of mutation were excluded from the analysis as the partitioning method utilised is most accurately applied to sequences with low levels of mutation.

### Partitioning of rearranged immunoglobulin sequences

The determination of genes and N regions of the sequences within the dataset was performed with the aid of a statistical analysis of point mutations [21]. This method uses the number of mutations in the core region of the V genes to predict the level of mutation within other regions of the immunoglobulin sequence. The approach is based upon the mutability of trinucleotides, while also factoring in the exponential decay of somatic point mutations [36] and the effects of antigen selection [21]. The key to the analysis is the calculation of mutability scores for the various genes. Mutability scores can be used to indicate the likelihood that mutations will be distributed in a particular way between two or more parts of an immunoglobulin sequence.

The focus of the study upon exonuclease removal required careful definition of rules for the identification of nucleotide losses. Preliminary alignment of sequences were performed using IMGT/V-QUEST [18]. Where identical IMGT/V-QUEST alignment scores were achieved to different alleles of a germline gene, the allele first allele reported was recorded. IMGT/V-QUEST compares input sequences to the IMGT reference sets which are the most complete sets of sequences that are available for IGHV, IGHD and IGHJ functional genes, their alleles and open reading frame genes. Alignments produced by IMGT/V-QUEST give an overall indication of similarity between a rearranged sequence and germline genes.

D gene determination was performed using previously described criteria [21], where the level of required similarity to a germline sequence was dictated by the length of the junction and the likelihood of N nucleotides being misidentified as IGHD segments by chance. To aid in the allocation of D genes, a D Gene Alignment Utility was developed. This web based tool allowed alignments to be performed between a junctional sequence and all germline D genes, including inverted D gene sequences [37] obtained from the IMGT Reference Directory [19]. The program utilized an altered Smith-Waterman algorithm that did not allow for gaps [38].

Difficulties with immunoglobulin partitioning are often experienced, especially in the determination of gene ends. In this study, runs of consecutive nucleotide differences between a IGH V-(D)-J rearrangement and a germline sequence at a gene end were always attributed to exonuclease removal, rather than mutation of the gene end. The portion of a IGH V-(D)-J rearrangement that contained such differences, with respect to the germline sequence, was allocated to the N region, and the gene was considered to have undergone exonuclease processing at the gene end.

Situations where IGHV or IGHJ ends revealed a series of differences and similarities to the germline sequence required the consideration of each possible combination of exonuclease removal and point mutation that could have led to the creation of the observed region end. Probabilities were calculated for each 'path' to the observed segment end. Mutability scores for the region end were used to determine the likelihood of point mutations contributing to the region end. TdT addition probabilities were used to calculate the likelihood of N additions generating particular nucleotide sequences. The TdT probabilities used were p(G) = 0.35, p(C) = 0.35, p(A) = 0.15, p(T) = 0.15 [5]. The path that displayed the greatest likelihood was used to allocate nucleotides to either an N region or a region end.

Sequences that lacked exonuclease removals from gene region end(s) were examined for the presence of P nucleotides [2]. Self-complementary repeats of the gene end located in the neighboring N region were designated as putative P nucleotides. For example, if the V gene ended with the nucleotides GA, then CT was sought at the start of the N1 region. All possible lengths of P insertions were considered as part of the identification process.

### Examination of P nucleotides

The contribution of P nucleotides to immunoglobulin diversity was investigated through data generated by the investigations reported here. Unique putative P nucleotides were identified and their appearance was tallied within the dataset. The number of junctions that displayed a lack of exonuclease activity at one or more gene ends was also calculated. The cumulative binomial probability of observing a given number of P nucleotides or greater was then calculated for each putative P nucleotide sequence in an attempt to estimate the contribution of P nucleotides to immunoglobulin diversity.

### Role of W and S Motifs in exonuclease processing

The effect of W and S motifs upon exonuclease activity was investigated within the IGHJ genes. The IGHJ genes were grouped based upon the relative location of such motifs. W motifs were defined as sequences of 3 or more consecutive A or T nucleotides. S motifs were defined as sequences of 2 or more consecutive G or C nucleotides. The presence of these motifs was considered within the first 15 5' nucleotides of the IGHJ genes. Three sets were established, based upon the observed configurations of the motifs in the IGHJ subgroups; S motif followed by W motif, S motif only and W motif only (Table 2). Analysis of exonuclease removals for each of these groups was then performed by calculating the average number of nucleotides removed from the IGHJ end for each set.

**Table 2:** Grouping of IGHJ genes by relative location of W and S motifs

J Gene	Sequence^1^	Group
IGHJ1*01	*GC*TGAATACTT*CC*AG	5' S only
IGHJ2*01	TGCTACT*GG*TACTTG	5' S only
IGHJ3*01	TGAT*GC*TTTTGATGT	5' S then W
IGHJ3*02	AT*GC*TTTTGATATCT	5' S then W
IGHJ4*01	ACTACTTTGACTACT	5' W only
IGHJ4*02	ACTACTTTGACTACT	5' W only
IGHJ4*03	*GC*TACTTTGACTACT	5' S then W
IGHJ5*01	ACAACT*GG*TT*CG*ACT	5' S only
IGHJ5*02	ACAACT*GG*TT*CG*ACC	5' S only
IGHJ6*01	ATTACTACTACTACT	5' W only
IGHJ6*02	ATTACTACTACTACT	5' W only
IGHJ6*03	ATTACTACTACTACT	5' W only

^1^W motifs shown underlined, S motifs shown in *italics*

### Statistical analysis

The extent of nucleotide deletion was calculated as the average number of nucleotides removed for a given dataset. Significant differences between average removals were determined using one-way ANOVA for normally distributed datasets, and Kruskal-Wallis Test for other datasets. Where significant differences were found, multiple comparison testing was carried out using Tukey's Multiple Comparison Test, for normally distributed datasets, and Dunn's Multiple Comparison Test for non-normally distributed datasets. All analysis of exonuclease removals was carried out using GraphPad Prism (Version 3.00, 1999, GraphPad Software) with an alpha value of 0.05.

An analysis of P nucleotides was performed by calculating the probability that putative P nucleotides may actually have resulted from N nucleotide addition by TdT. Probabilities were calculated as described by Meier and Lewis [8]. The probability of the presence of the observed or a greater number of P nucleotides was calculated as follows [8]:



where, *n *is the observed number of P nucleotides and *N *is the total number of sequences containing junctional inserts equal to or greater than the length of the P nucleotide(s) being examined, and *p *is the expected frequency of the observed P nucleotide sequence, which was calculated using reported TdT frequencies [5]. Probabilities were calculated for each observed putative P nucleotide sequence, and the alpha value was adjusted for the number of comparisons made, using the Bonferroni correction.

## Authors contributions

KJ carried out the dataset creation and partitioning, performed the statistical analysis and drafted the paper. BG aided in the design of the study and the development of the statistical analysis. WS advised on study design and development. AC devised the study, aided in study design and co-ordination and revised the manuscript. All authors read and approved the final manuscript.

## Supplementary Material

Additional File 1 **294 Partitioned human IgM Sequences **294 human IgM sequences were partitioned using a statistically based model and used in the examination of exonuclease activity and P nucleotide addition in the expressed human repertoire. The file is in Microsoft Excel format. Click here for file
